# Dose-response relationship between exercise and cognitive function in older adults with and without cognitive impairment: A systematic review and meta-analysis

**DOI:** 10.1371/journal.pone.0210036

**Published:** 2019-01-10

**Authors:** Lianne M. J. Sanders, Tibor Hortobágyi, Sacha la Bastide-van Gemert, Eddy A. van der Zee, Marieke J. G. van Heuvelen

**Affiliations:** 1 Center for Human Movement Sciences, University of Groningen, University Medical Center Groningen, Groningen, The Netherlands; 2 Department of Epidemiology, University of Groningen, University Medical Center Groningen, Groningen, Netherlands; 3 Groningen Institute for Evolutionary Life Sciences (GELIFES), University of Groningen, Groningen, The Netherlands; EHESP Paris, FRANCE

## Abstract

This systematic review and meta-analysis examined the dose-response relationship between exercise and cognitive function in older adults with and without cognitive impairments. We included single-modality randomized controlled aerobic, anaerobic, multicomponent or psychomotor exercise trials that quantified training frequency, session and program duration and specified intensity quantitatively or qualitatively. We defined total exercise duration in minutes as the product of program duration, session duration, and frequency. For each study, we grouped test-specific Hedges’ *d* (n = 163) and Cohen’s *d* (n = 23) effect sizes in the domains Global cognition, Executive function and Memory. We used multilevel mixed-effects models to investigate dose-related predictors of exercise effects. In healthy older adults (n = 23 studies), there was a small positive effect of exercise on executive function (d = 0.27) and memory (d = 0.24), but dose-parameters did not predict the magnitude of effect sizes. In older adults with cognitive impairments (n = 13 studies), exercise had a moderate positive effect on global cognition (d = 0.37). For older adults with cognitive impairments, we found evidence for exercise programs with a short session duration and high frequency to predict higher effect sizes (d = 0.43–0.50). In healthy older adults, dose-parameters did not predict the magnitude of exercise effects on cognition. For older adults with cognitive impairments, exercise programs with shorter session duration and higher frequency may generate the best cognitive results. Studies are needed in which different exercise doses are directly compared among randomized subjects or conditions.

## Introduction

The number of dementia patients may triple to 135M globally by 2050 [1]. Dementia is characterized by a progressive decline in neurocognitive function. Pharmacological treatments may moderate symptoms but can cause adverse effects [2]. Exercise might be an effective and safe alternative to drugs to slow cognitive decline. Exercise may improve certain cognitive functions in old age by inducing the release of brain-derived neurotrophic factor (BDNF [3,4]) and insulin-like growth factor-1 (IGF-1 [5,6]), thereby potentially facilitating structural and connectivity changes in the hippocampus, temporal lobe, frontal areas and corpus callosum [7–11], structures that are activated during tasks requiring executive function, attention, processing speed and memory.

Exercise type and dose-parameters may determine the magnitude of effects on cognition and how long these effects persist after an intervention [12,13]. Dose-parameters include program duration (number of weeks), session duration (length of each session in minutes including warm-up and cool-down), frequency (session rate per week) and intensity. Exercise intensity refers to the amount of effort or energy that is required to perform a physical activity [14] and is often expressed as percentage of maximal oxygen update (VO2max) required during a physical activity [15].

High compared with low exercise dose-parameters tend to predict better physical fitness outcomes in older adults. Meta-analyses revealed that longer program duration [16–18] and higher intensity [16,18] were associated with gains in muscle strength and VO2max of older adults. Program duration also correlated with gains in endurance, lower extremity muscle strength, balance and levels of Activities of Daily Living (ADL) in older subjects with dementia [19]. Exercise intensity was related to improvements in fitness- and health-related parameters such as VO2max and mortality in healthy middle-aged and older adults [20–25]. Exercise-induced improvements in physical fitness may facilitate brain plasticity and secondarily improvements in cognitive function through increases in brain activation. Indeed, higher cardiorespiratory fitness [26,27] and exercise-induced adaptions in blood lactate [28] were previously associated with higher brain activation in anterior and motor areas [26,27], fronto-cingulo-parietal networks [28] and better executive performance [26,27]. Considering that exercise dose-parameters are related to increases in fitness, and fitness increases may in turn be related to cognitive function by facilitating brain plasticity, exercise dose-parameters may be related to increases in cognitive functions. Indeed, in healthy young and older adults, high dose-parameters of acute exercise were related to gains in executive function such as processing speed and inhibitory control [29–32]. Exercise-induced cognitive benefits of acute exercise may accumulate to greater and lasting cognitive improvements with chronic exercise in a dose-specific way.

The relationship between exercise dose-parameters and cognitive functions in chronic exercise studies is still not fully understood. A meta-analysis suggested that exercising for 45–60 minutes per session, at least at moderate intensity, and at the highest feasible frequency can improve global cognition, attention, executive function and (working) memory in healthy adults over 50 [33], but the authors did not examine total dose. A meta-analysis of 18 randomized controlled trials (RCTs) [34] showed that weekly exercise duration (≤150 or >150 minutes) was not related to changes in cognitive function in older adults with cognitive impairments, specifically Alzheimer’s disease (AD) and non-AD dementia, but other dose-parameters were not investigated. There is thus a need to systematically review whether improvements in cognitive function scale with exercise dose-parameters separately and as total dose and if dosing effects vary with cognitive status. The aim of the present review was to examine the relationship between exercise dose-parameters (program and session duration, frequency, intensity) and cognitive function (global cognition, executive function, memory) in adults with vs. without cognitive impairments. We quantified the dose-response relationship separately between the responses to aerobic, anaerobic, multimodal, and psychomotor interventions and changes in global cognition, executive function, and memory using advanced statistical modeling. We hypothesized that the magnitude of exercise effects on global cognition, executive function, and memory is related to exercise dose-parameters separately or combined as total dose. The results of the present study can be used to update exercise recommendations and implement exercise programs for older adults with and without cognitive impairments.

## Methods

The current protocol is registered with the Open Science Framework (url: https://osf.io/qe43p/). PRISMA guidelines were followed [35] (S1 Checklist).

### Search strategy and selection criteria

We searched databases PubMed, Embase, Psycinfo, Web of Science and the Cochrane Central Register of Controlled Trials from inception (database-specific onset date) through December 4^th^, 2017. We included human studies that were Randomized Controlled Trials (RCTs). Specific Emtree (Embase) and MeSH (PubMed) terms included exercise, cognition, memory and executive function. Nonspecific terms represented activity, training type, cognitive outcome and study design. We filtered studies with children, adolescents or patient populations other than Mild Cognitive Impairment (MCI), Vascular Cognitive Impairment (VCI) or dementia, protocol papers and virtual designs. S1 Table lists the search terms.

Two authors (LS and MvH) independently selected articles for inclusion by screening the titles and abstracts (95.3% agreement) resulting in full-text screening of the articles in question. Full-text screening was done for all articles (LS) selected by either one of the authors. Lastly, we handsearched reviews for relevant articles (LS).

### Inclusion- and exclusion criteria

We included studies that satisfied the following criteria: (1) participants were aged ≥18 years, (2) participants were healthy or diagnosed with MCI, VCI or dementia, (3) the intervention consisted of aerobic, anaerobic, multicomponent or psychomotor exercise of any intensity or frequency, and a duration of ≥4 weeks (as meaningful improvements are believed to appear after ≥4 weeks of exercise [36]), (4) the exact range of frequency and session duration was specified, (5) the training intensity was specified descriptively (e.g., ‘moderate intensity’) or objectively, (6) there was a cognitive outcome measure measured by neuropsychological tests. Studies were excluded if: (1) the physical intervention included a non-physical component and (2) the control group performed non-contrasting activities (contrasting activities include non-physical activity or stretching and toning).

### Data extraction

We extracted the following data from the included studies: sample characteristics (sample size, age, gender, education, cognitive health status), intervention parameters (exercise mode, program and session duration, frequency and intensity) and outcome measures (means, standard deviations or F-values of the cognitive tests at baseline and post-intervention). If necessary, the original authors were contacted for any missing data.

### Dose

We calculated exercise duration in minutes for every study using the program duration (weeks), session duration (minutes) and frequency. We averaged frequency, total session duration and intensity measures if necessary (i.e., if sessions lasted 30–40 minutes, we used 35 minutes as average).

For aerobic and psychomotor exercise, intensity was expressed as % maximum heartrate (HRmax), % heart rate reserve (HRR), or % maximum oxygen update (VO2max). For anaerobic exercise, we multiplied the target number of sets and repetitions with the training intensity in % one repetition maximum (1RM, the maximum amount of weight that a person can lift once) or VO2max. For multicomponent exercise, we calculated the average intensity of the aerobic and anaerobic intensity coefficients if both could be calculated from the data.

If intensity was given descriptively or in terms of the rate of perceived exertion (RPE Borg scale [37]), we took the corresponding heart rate in accordance with the American College of Sports Medicine guidelines [36]. We set ‘light’ intensity as 30–40% HRR/1RM, ‘moderate’ intensity as 60–80% of HRR/1RM and ‘high’ intensity as 80–100% HRR/1RM.

### Effect size

We calculated Hedges’ *g* effect sizes (ESs) for each cognitive outcome. We subtracted the mean change (post-pre) in the control group from the mean change in the exercise group and divided this difference by the pooled standard deviation of the baseline scores [38]. We obtained Hedges’ *d* by adjusting Hedges’ *g* for small sample size bias [39].

If means and standard deviations could not be retrieved from the text, we retrieved the F statistic for the Group x Time interaction and used it to calculate Cohen’s *d*:

$$\text{Cohen}\prime \text{s}\;\text{d}=\sqrt{\text{F}\left[\left(\frac{{\text{n}}_{\text{exp}}+{\text{n}}_{\text{cont}}}{{\text{n}}_{\text{exp}}*{\text{n}}_{\text{cont}}}\right)*\left(\frac{{\text{n}}_{\text{exp}}+{\text{n}}_{\text{cont}}}{{\text{n}}_{\text{exp}}+{\text{n}}_{\text{cont}}-2}\right)\right]}$$

We also adjusted Cohen’s *d* for small sample size bias. When a lower test score represented better performance, we multiplied Hedges’ (n = 163) and Cohen’s (n = 23) *d* with -1, so that a positive *d* always indicates better performance in the exercise group. We considered Hedges’ and Cohen’s *d* = 0.2, *d* = 0.5 and *d* = 0.8 as, respectively, small, medium and large effect sizes [40].

We grouped the effect sizes of the cognitive tests in 1) global cognition, 2) executive function, or 3) memory. We identified tests as falling in one of these domains by using the categorization of the respective authors (i.e., if a test was described as a global cognitive test, we grouped the test in global cognition). When a test was described as memory test in some papers but as executive function test in others (e.g., working memory tests), we used the categorization that most authors adhered to. For this reason, two authors (LS and MvH) decided to group working memory tests within the executive function domain. Appendices 4a and 4b list the tests that were grouped in each domain for every study. We excluded cognitive tests that could not be grouped within our domains (e.g., reading ability or visuospatial ability). 95% Confidence intervals for the average ESs were calculated with the formula

95%CI=ES±1.96*SE

where SE is the standard error:

$$SE=\sqrt{(\frac{{\text{n}}_{\text{exp}}+{\text{n}}_{\text{cont}}}{{\text{n}}_{\text{exp}}*{\text{n}}_{\text{cont}}})+\frac{d^{2\;}}{2*({\text{n}}_{\text{exp}}+{\text{n}}_{\text{cont}}-2)}}$$

[39].

### Study quality

One author (LS) evaluated the quality of the included studies using the 11-item Physiotherapy Evidence Database (PEDro) scale [41]. The PEDro scale rates the methodological quality of a study based off of randomization, allocation, blinding, analyses and reporting of outcomes. Scores ≤3 indicate poor study quality, 4–5 fair quality and 6–10 good to excellent quality.

### Statistical analyses

We computed means and standard deviations. We used the SPSS MeanES macro ([42,43], SPSS 23.0, IBM, Armonk, NY) to generate mean effect sizes and indices of heterogeneity (Cochran’s Q, I^2^) [44] for the forest plots, and S2 and S3 Tables. I^2^ values<0.25 were indicative of limited heterogeneity, 0.25<I^2^<0.50 indicated moderate heterogeneity and I^2^>0.50 large heterogeneity [44]. We used R version 3.4.3 (R Core Team, 2013) and the R Metafor package [45] to analyze the data and set two-tailed significance at p≤0.05. Publication bias was evaluated with a funnel plot (S1 Fig) [46]. To analyze the effects of multiple moderators on ESs, we used multilevel mixed effects (with effect size ID and study ID as random effects) models using restricted maximum likelihood estimation [47,48]. Such models account for dependencies between test-specific effect sizes by taking into account the nesting of multiple effect sizes within studies. We used mixed-effects models to examine: 1) differences in exercise effects between healthy older adults with and without cognitive impairments; 2) differences in exercise effects for global cognition vs. executive function and memory; 3) differences in exercise effects across the four exercise types (aerobic, anaerobic, multimodal, psychomotor); 4) associations between a) total exercise duration and b) intensity and cognitive effects, and 5) associations between the separate dose-parameters (program duration, session duration, frequency) and cognitive effects.

## Results

### Study characteristics

**Fig 1:**
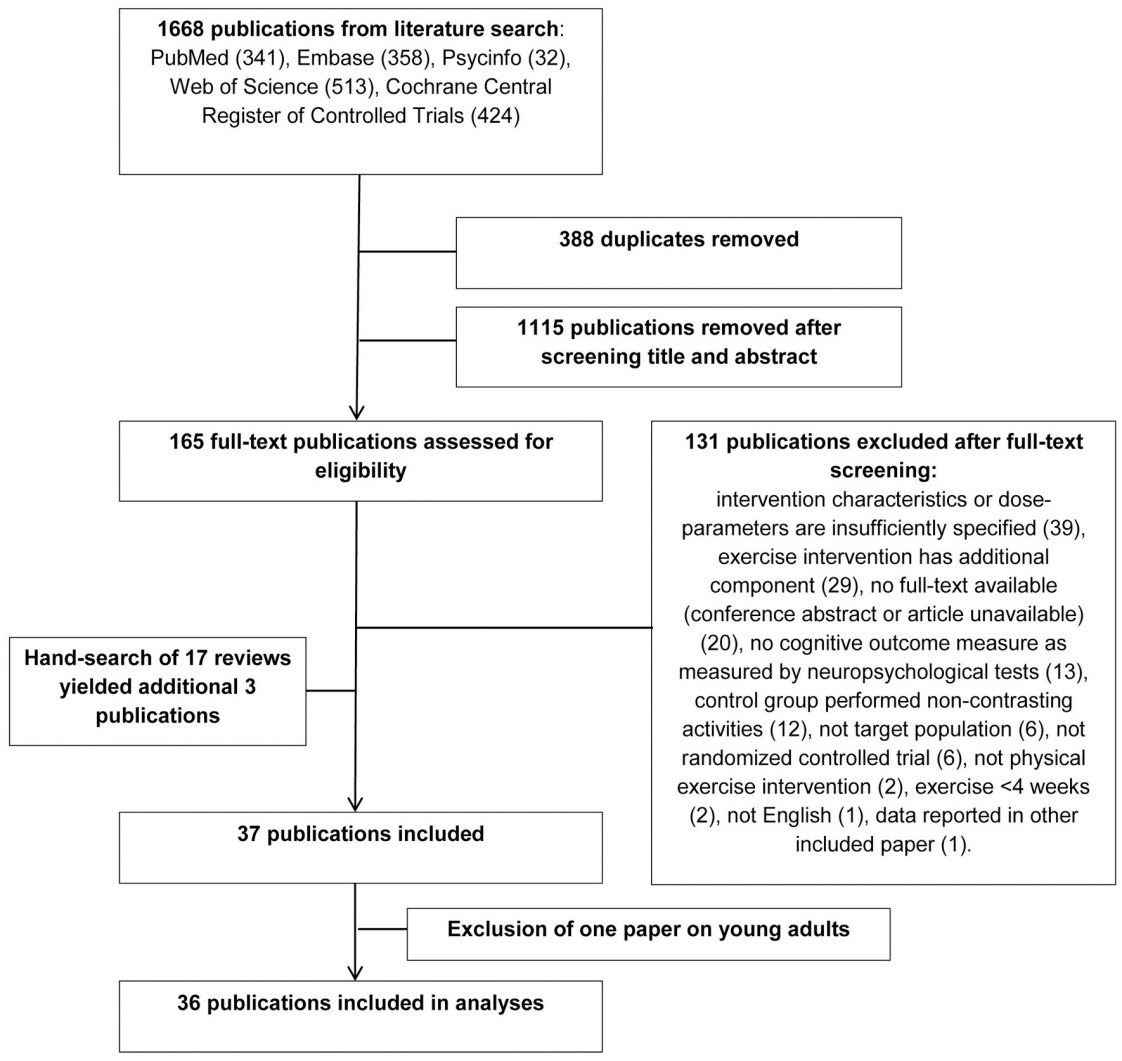
Study selection process.

Fig 1 depicts the selection process. A total of 37 studies were eligible for inclusion [49–86]. As 36 of the 37 studies included older populations (>50), we excluded one study with young adults [86] to facilitate comparison. Tables 1 and 2 list the included studies.

**Table 1:** Study characteristics for included studies with healthy older adults.

Author, year	N (#♀) Intervention/control	Age (M±SD) Intervention/control	Baseline MMSE (M±SD) Intervention/control	Intervention type (ar/an/mc/pm)^a^	Intervention activities	Program duration (weeks), session duration (minutes), frequency(#/wk)	Exercise intensity	Control
Albinet et al., 2010	12(6) / 12(7)	70.9±4.9 / 70.4±3.4	28.5±1.1 / 29±0.9	ar	walking, circuit-training, step, gradually running	12,60,3	40–60% HRR+HRrest	stretching
Albinet et al., 2016	19(13) / 17(13)	67±5 / 66±5	29.1±1.1 / 28.7±1.5	ar	aerobics, swimming	21,60,2	40–65% HRR	stretching
Ansai & Rebelatto, 2015	23(17) / 23(15) 23(15) / 23(15)	81.9±1.9 / 82.6±2.6 82.8±2.8 / 82.6±2.6	24.3±3.3 / 25.3±3.5 25.1±3.4 / 25.3±3.5	mc an	cycle ergometer & strength exercises of major muscle groups with extra loading leg press, chest press, calf, back extension, abdominal and rowing	16,60,3	AR: 60–85% of HRR, AN: 70–80% of 1RM corresponding to RPE 14–17, 2 sets of 1–15 repetitions 90–100% 1RM as referred by authors, 3 sets of 10–12 repetitions	non-active
		……‥	……‥					
Best et al., 2015	54♀♀ / 49♀♀	69.5±2.7 / 70.0±3.3;	28.5±1.3 / 28.8±1.2;	an	resistance training with machine and non-machine exercises	52,60,1	80–100% of 1RM corresponding to ‘high intensity’, 2 sets of 6–8 repetitions	balance and tone
	52♀♀ / 49♀♀	69.4±3.0 / 70.0±3.3	28.6±1.5 / 28.8±1.2	an	resistance training with machine and non-machine exercises	52,60,2	80–100% of 1RM corresponding to ‘high intensity’, 2 sets of 6–8 repetitions	balance and tone
Coetsee & Terblanche, 2017	13(10) / 19(11)	64.5±6.3 / 62.5±5.6	MoCA: 27.9±1.5 / 28.2±1.6	ar	high-intensity interval treadmill walking	16,30,3	4 intervals of 4 minutes at 90–95% HRmax, interspersed by 3 minutes active recovery at 70% HRmax	no exercise
	13(10) / 19(11)	61.6±5.8 / 62.5±5.6	MoCA: 27.6±1.3 / 28.2±1.6	ar	continuous treadmill walking	16,47,3	70–75% Hrmax	no exercise
	22(15) / 19(11)	62.4±5.1 / 62.5±5.6	MoCA: 27.5±1.3 / 28.2±1.6	an	upper and lower body resistance exercises using machines and free weights	16,30,3	50–100% of 10RM, 10 sets of 10 repetitions	no exercise
Dao et al., 2013	37♀♀ / 36♀♀	69.2±2.6 / 69.8±3.2;	28.8±1.2 / 28.8±1.2;	an	resistance training with machine and non-machine exercises	52,60,1	80–100% of 1RM corresponding to ‘high intensity’, 2 sets of 6–8 repetitions	balance and tone
	41♀♀ / 36♀♀	69.4±3.0 / 69.8±3.2	28.5±1.6 / 28.8±1.2	an	resistance training with machine and non-machine exercises	52,60,2	80–100% of 1RM corresponding to ‘high intensity’, 2 sets of 6–8 repetitions	balance and tone
Fabre et al., 2002	8(6)/ 8(7)	65.4±2.2 / 65.7±1.5	nr^b^	ar	walking and running	8,60,2	Ventilatory threshold corresponding to 50–70% HRmax	leisure activities
Ferreira et al., 2015	22(15) / 22(19)	66.2±5.6 / 69.2±4.8	28.8±1.6 / 28.7±1.5	ar	supervised walking	24,40–50,3	60–80% HRR	social interaction
Iuliano et al., 2015	20(11) / 20(12)	65.80±6.32 / 66.47±6.32	nr^b^	an	whole-body strength exercises on isotonic machines	12,30,3	60–85%1RM, 3 sets of 6–12 repetitions	non-active
	20(12) / 20(12)	68.44±6.40 / 66.47±6.32	nr^b^	ar	cardiovascular training on ergometer machines	12,30,3	50–80% HRR	non-active
	20(13) / 20(12)	66.67±5.83 / 66.47±6.32	nr^b^	pm	postural and balance exercises	12,40,3	30–40% HRmax corresponding to ‘low intensity’ exercise	non-active
Jonasson et al., 2017	29(15) / 29(17)	68.4±2.54 / 69.0±2.91	28.7±1.16 / 29.5±0.64	ar	walking or jogging, cycling or training on cross-trainer	24,30–60, 3	40–80% HRmax	stretching and toning
Kimura et al., 2010	65(40) / 54(30)	73.6±4.7 / 75.2±6.3	27.8±1.8 / 27.9±2.1	an	progressive resistance and balance training	12,90,2	60–100% of 1RM corresponding to ‘moderate to high intensity’, 2–3 sets of 10 repetitions	health education
Liu-Ambrose et al., 2010	54♀♀ / 49♀♀	69.5±2.7 / 70.0±3.3	28.5±1.3 / 28.8±1.2	an	resistance training with machine and non-machine exercises	52,60,1	80–100% of 1RM corresponding to ‘high intensity’, 2 sets of 6–8 repetitions	balance and tone
	52♀♀ / 49♀♀	69.4±3.0 / 70.0±3.3	28.6±1.5 / 28.8±1.2	an	resistance training with machine and non-machine exercises	52,60,2	80–100% of 1RM corresponding to ‘high intensity’, 2 sets of 6–8 repetitions	balance and tone
Liu-Ambrose et al., 2012	20♀♀ / 17♀♀	69.7±2.8 / 69.2±3.2	28.6±1.2 / 29.1±1.1	an	resistance training with machine and non-machine exercises	52,60,1	80–100% of 1RM corresponding to ‘high intensity’, 2 sets of 6–8 repetitions	balance and tone
	15♀♀ / 17♀♀	68.9±3.2 / 69.2±3.2	29.1±0.8 / 29.1±1.1	an	resistance training with machine and non-machine exercises	52,60,2	80–100% of 1RM corresponding to ‘high intensity’, 2 sets of 6–8 repetitions	balance and tone
Maass et al., 2015	21(11) / 19(11)	68.8±4.5 / 67.9±4.1	28.95±0.92 / 28.95±0.91	ar	interval training on stationary treadmills	12,40,3	65–70% HRR	progressive muscle relaxation/stretching
Madden et al., 1989	25(11) / 26(14)	66.52±4.07 / 66.62±3.54	nr^b^	ar	supervised bicycle ergometry, walking/jogging	16,60,3	70% HRR	wait list
Muscari et al., 2010	60(28) / 60(30)	68.8±2.5 / 69.9±2.8	26.7±nr/27±nr	ar	cycle ergometer, treadmill and free-body activity	52,60,3	70% HRmax	lifestyle education
Nouchi et al., 2013	32(nr**) / 32(nr**)	66.75±4.61 / 67.06±2.82	27.91±1.25 / 27.94±1.27	mc	combination aerobic, strength and stretching exercise	4,30,3	60–80% HRmax	wait list
Ruscheweyh et al., 2011	20(14) / 21(14)	60.1±6.2 / 58.1±6.7	29.5±0.8 / 29.3±0.8	ar	nording walking	24,50,3–5	50–60% maximal exertion	non-active
	21(13) / 21(14)	62.5±6.4 / 58.1±6.7	29.2±0.8 / 29.3±0.8	pm	stretching, limbering and toning of upper and lower extremities	24,50,3–5	30–40% maximal exertion	non-active
Shatil et al., 2013	31(22) / 29(19)	79±5.76 / 81±5.25	nr^b^	mc	cardiovascular workout seated and standing, strength training, flexibility training	16,45,3	30–40% HRmax corresponding to ‘low intensity exercise’	book club
Tsai et al., 2015	24♂♂ / 24♂♂	70.79±3.39 / 72.00±4.14	27.88±1.19 / 28.21±0.98	an	core exercises with machines and free weights	52,60,3	75–80% of 1RM, 3 sets of 10 repetitions	non-active
Tsai et al., 2017	21♂♂ / 21♂♂	66.2±4.9 / 65.7±3.5	27.5±3.03 / 27.7±1.80	ar	cycling on bicycle ergometer or walking on treadmill	24,40,3	70–75% HRR	balance and stretching
Tsutsumi et al., 1997	13(3) / 14(3)	67.8±4.9 / 69.8±4.6	nr^b^	an	major muscle exercises with weight machines	12,20–34,3	75–85% of 1RM, 2 sets of 8–10 repetitions	no exercise
	14(3) / 14(3)	68.9±7.5 / 69.8±4.6	nr^b^	an	major muscle exercises with weight machines	12,34–60,3	55–65% of 1RM, 2 sets of 14–16 repetitions	no exercise
Vedovelli et al., 2017	20♀♀ / 9♀♀	83.0±6.5 / 77.3±9.9	24.1±3.30 / 24.8±3.30	mc	walking, upper and lower body strengthening exercises	12,60,3	75–85% HRmax, 50–75% 1RM, 3 sets of 10 repetitions	non-active

^a^Ar = aerobic; an = anaerobic; mc = multi-component; pm = psychomotor.

^b^nr = not reported.

**Table 2:** Study characteristics for included studies with older adults with cognitive impairments.

Author, year	Population^a^	N (#♀) Intervention/control	Age (M±SD) Intervention/control	Baseline MMSE (M±SD) Intervention/control	Intervention type^b^ (ar/an/mc/pm)	Intervention activities	Program duration (weeks), session duration (minutes), frequency(#/wk)	Exercise intensity	Control
Baker et al., 2010	MCI	19(10) / 10(5)	65.3±9.4**♀**; 70.9±6.7♂ / 74.6±11.1♀; 70.6±6.1♂	28.4±1.7♀; 25.6±2.4♂ / 28.6±1.7♀; 27.2±1.8♂	ar	treadmill, stationary bicycle, elliptical trainer	24,45–60,4	(gradual increase to) 75–85% HRR	stretching
Bossers et al., 2015	dementia	37(29) / 36(25)	85.7±5.1 / 85.4±5.0	15.8±4.3 / 15.9±4.2	mc	walking + lower-limb strength exercises	9,30,4	AR: 50–85% HRmax; AN: 60–100% 1RM corresponding to RPE>12, 2 sets of 6–12 repetitions	social visits
		36(28) / 36(25)	85.4±5.4 / 85.4±5.0	15.2±4.8 / 15.9±4.2	ar	walking	9,30,4	50–85% HRmax	social visits
Ten Brinke et al., 2015	probable MCI	14♀♀ / 13♀♀	76.1±3.4 / 75.5±3.9	27.5±1.51 / 27.2±1.9	ar	outdoor walking	24,60,2	RPE 13–15, 40–80% HRR	balance and tone
		12♀♀ / 13♀♀	73.8±3.7 / 75.5±3.9	26.7+2.6 / 27.2±1.9	an	whole-body strength exercises with and without machines	24,60,2	60–80% 1RM corresponding to RPE 13–15, 2 sets of 6–8 repetitions	balance and tone
Davis et al., 2013	probable MCI	30♀♀ / 28♀♀	75.5±3.5 / 75.0±3.7	MoCA: 22.2+2.8 / 22.5±2.8	ar	outdoor walking	24,60,2	40–60% HRR	balance and tone
		28♀♀ / 28♀♀	74.1±3.6 / 75.0±3.7	MoCA: 21.4+3.4 / 22.5±2.8	an	whole-body strength exercises with and without machines	24,60,2	80–100% of 1RM corresponding to ‘high intensity’, 2 sets of 6–8 repetitions	balance and tone
De Souto Barreto et al., 2017	dementia	44(41) / 47(36)	88.3±5.1 / 86.9±5.8	11.4±6.2 / 10.8±5.5	mc	aerobic, coordination en strengthening exercises	24,60,2	60–80% of HRR/1RM	social activites
Kemoun et al., 2010	dementia	16(12) / 15(11)	82.0±5.8 / 81.7±5.1	12.6 / 12.9	mc	exercises in walking, equilibrium, stamina	15,60,3	60–70% HRR	non-active
Kwak et al., 2006	dementia	15♀♀ / 15♀♀	79.67±6.64 / 82.27±7.09	14.53±5.34 / 13.47±7.04	an	chair exercise + stretching	52,30–40,2–3	30–60% VO2max + 2–3 sets of 2–20 repetitions with 7–8 exercises	nr ^c^
Liu-Ambrose et al., 2016	VCI	35(19)/ 35/(17)	74.8±8.4 / 73.7±8.3	26.3±2.7 / 26.4±3.1	ar	walking	24,60,3	40–60% HRR	usual care + education
Nagamatsu et al., 2013	probable MCI	30♀♀ / 28♀♀	75.6±3.6 / 75.1±3.6	27.4±1.5 / 27.1±1.7	ar	outdoor walking	26,60,2	RPE 13–15, 40–80% HRR	balance and tone
		28♀♀ / 28♀♀	73.9±3.4 / 75.1±3.6	27.0+1.8 / 27.1±1.7	an	whole-body strength exercises with and without machines	26,60,2	60–80% 1RM corresponding to RPE 13–15, 2 sets of 6–8 repetitions	balance and tone
Ruiz et al., 2015	cognitive impairments	20(16) / 20(16)	92.3±2.3 / 92.1±2.3	18.5±6.2 / 16.4±6.5	mc	aerobic exercise on cycle-ergometer and lower-limb strength exercises	8,40–45,3	30–40%HRmax corresponding to Borg RPE 10–12, 30–70%1RM, 2–3 sets of 6–8 repetitions	standard care
Telenius et al., 2015	dementia	87(63) / 83(62)	87.3±7.0 / 86.5±7.7	15.5±0.6 / 15.7±4.9	mc	strength, balance, and gait exercises	12,50–60,2	12RM, 80–100% of 1RM corresponding to ‘high intensity’,	stretching and relaxing activities
Varela et al., 2011	MCI	17(nr**) / 15(nr**)	79.24±10.07 / 79.40±6.72	19.86±5.12 / 21.80±3.23	ar	lower intensity cycling in recumbent bike	12,30,3	40% HRR	recreational activities
		16(nr**) / 15(nr**)	76.44±11.38 / 79.40±6.72	20.81±4.69 / 21.80±3.23	ar	higher intensity cycling in recumbent bike	12,30,3	60% HRR	recreational activities
Wei & Ji, 2014	MCI	30(9) / 30(11)	66.73±5.48 / 65.27±4.63	24.33±1.65 / 25.00±1.29	mc	handball exercises	24,30,5	60% HRmax	original life entertainment

^a^MCI = mild cognitive impairment; VCI = vascular cognitive impairment.

^b^ar = aerobic; an = anaerobic; mc = multi-component; pm = psychomotor.

^c^nr = not reported.

In total, there were 2007 participants (1772 women). If studies reported different test results of exactly the same samples, we nested the test-specific effect sizes within one study. Studies that only used parts of the same samples were treated as separate studies in the analyses. Although we acknowledge that this induces some non-independence, this method allows for effect sizes to be paired with the correct sample characteristics and sample size. The mean age weighted for sample size was 72.8±6.57 years.

The funnel plot (S1 Fig) revealed some publication bias. Egger’s test was indicative of significant asymmetry (bias = 1.77 (95%CI 0.72–2.81)). The asymmetry was partly due to three studies [64,68,85] which yielded effect sizes >1 with moderate to small sample sizes (respectively n = 60, n = 32, n = 31). These studies remained included in the analyses.

There was a small inverse weighted correlation between higher PEDro score and lower ESs (r = -0.200, p≤0.01).

Tables 3 and 4 show the descriptive statistics for the dose-parameters, total exercise duration (minutes) and intensity (arbitrary unit (a.u.), for details see paragraph *Dose* in Methods). S4 and S5 Tables list the correlations weighted for sample size per study between dose-parameters and sociodemographic factors. Program duration, session duration and frequency highly correlated for all older adults, with longer programs often having longer but fewer sessions/week.

**Table 3:** Weighted^a^ descriptive statistics for dose-parameters.

	Healthy	Impaired	Total
Mean # participants (SD)	49.4 (22.7)	54.3 (23.6)	50.8 (23.0)
Mean age (SD)	70.3 (5.32)	78.3 (5.59)	72.8 (6.57)
Mean MMSE (SD)	27.7 (2.49)	22.9 (5.64)	25.4 (5.00)
Mean program duration in weeks (SD)	22.9 (16.5)	21.1 (7.87)	22.3 (14.4)
Mean session duration in minutes (SD)	49.3 (14.3)	52.0 (12.8)	50.1 (13.9)
Mean frequency (#/week, SD)	2.63 (0.67)	2.60 (0.88)	2.62 (0.75)
Mean total exercise duration in minutes (SD)	2752.4 (1992.1)	2647.8 (1053.2)	2720.0 (1761.7)

^a^Descriptive statistics weighted for n per study.

**Table 4:** Weighted^a^ descriptive statistics for total exercise duration and intensity per exercise category.

		Aerobic	Anaerobic	Multicomponent	Psychomotor
Mean total exercise duration in minutes (SD)	Healthy	2974.0^b^ (1896.7)	3627.8^c^ (2232.0)	1520.1^c^ (938.1)	1940.4^b^ (1198.4)
	Cognitive impairments	2822.8^b^ (1109.5)	3155.6^c^ (277.3)	1670.1^c^ (880.1)	-
Mean intensity^b^^c^ (SD)	Healthy	65.2^b^ (7.65)	1655.0^c^ (528.3)	198.8^c^ (316.0)	35.0^b^ (n/a)
	Cognitive impairments	58.9^b^ (5.45)	1112.6^c^ (68.9)	514.0^c^ (384.3)	-

^a^Descriptive statistics weighted for n per study.

^b^%HRR/HRmax/VO2max.

^c^a.u. arbitrary unit.

### Dose-response association of exercise on cognition in healthy older populations

The 23 studies in this category included 1225 participants (1134 women). Mean age was 70.3±5.32 and mean Mini Mental State Examination (MMSE) score was 27.7±2.49 (Table 1).

**Fig 2:**
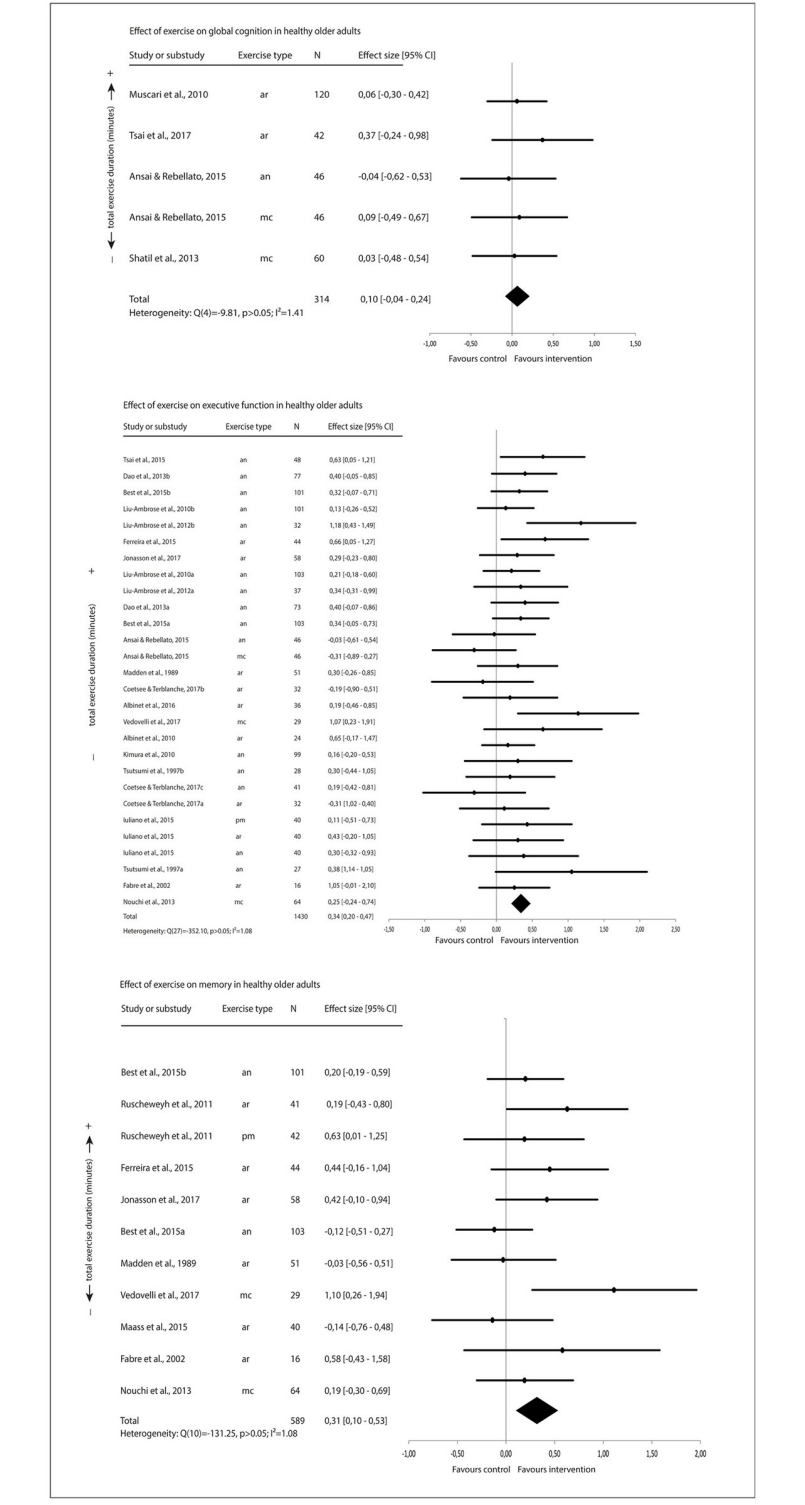
Effect of exercise on cognitive function in healthy older adults.

The forest plots (Fig 2) show a small positive exercise effect on averaged executive function and averaged memory but not on averaged global cognition.

### Dose-response association of exercise on cognition in older populations with cognitive impairments

There were 13 studies in older populations with cognitive impairments with varying etiologies (Table 2). In total, there were 782 participants (676 women). The mean age was 78.3±5.64and mean MMSE score was 22.9±5.64.

**Fig 3:**
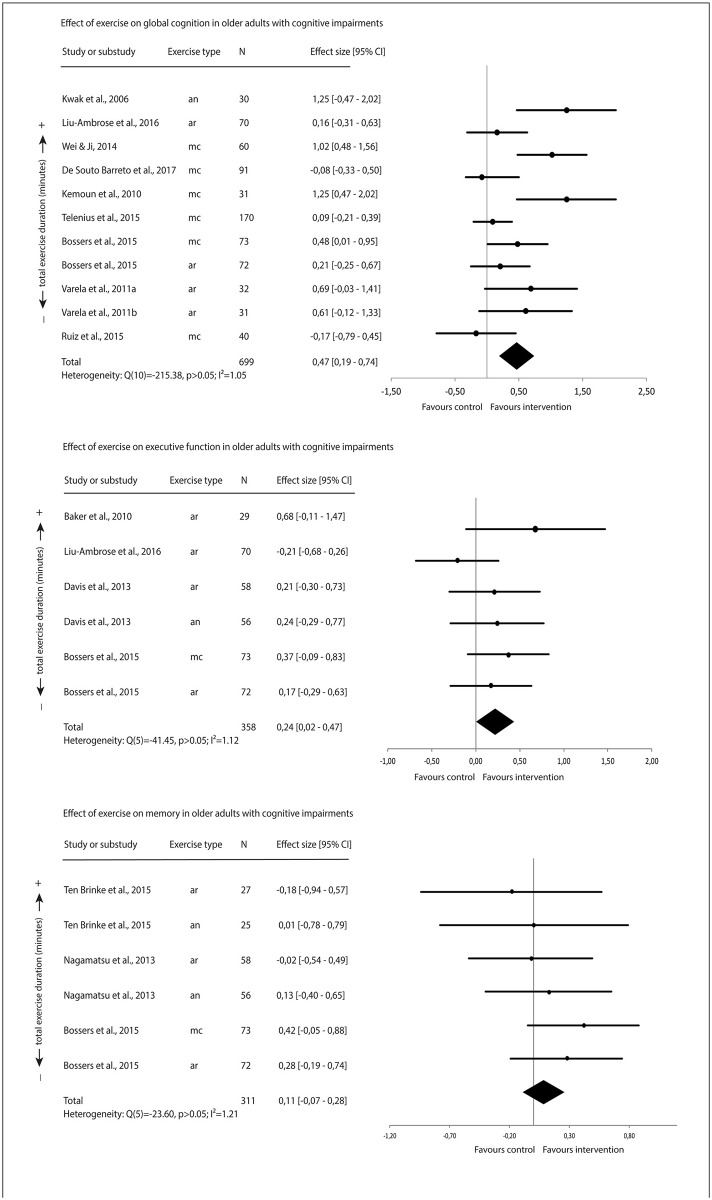
Effect of exercise on cognitive function in older adults with cognitive impairment.

Fig 3 displays the forest plots of the averaged effect sizes. There was a moderate positive effect of exercise on averaged global cognition, and a small positive effect of exercise on averaged executive function. There was no evidence for a significant effect of exercise onaveraged memory.

### Mixed-effects models

Mixed-effects models revealed a significant but small overall effect size of d = 0.24 (p≤0.001). The variation in test-specific effect sizes within vs. between studies was respectively 4.8% (p>0.05) and 41.4% (p≤0.001).

Table 5 shows the outcomes for the moderator analyses. For older adults with and without cognitive impairments combined, there were no significant predictors of effect size (healthy, cognitive impairments; F(1,184) = 0.168, p>0.05), cognitive domain (global cognition, executive function, memory; F(2,183) = 0.870, p>0.05), type of exercise (aerobic, anaerobic, multicomponent, psychomotor; F(3,182) = 0.322, p>0.05), program duration (4–12 weeks, 13–24 weeks, >24 weeks; F(2,137) = 1.157, p>0.05), session duration (0–30 minutes, 31–45 minutes, >45 minutes; F(2,183) = 0.816, p>0.05); frequency (1/week, 2/week, 3/week, ≥4/week; F(3,182) = 1.242, p>0.05); total exercise duration (F(1,184) = 0.302, p>0.05), intensity (aerobic training: F(2,77 = 2.396, p>0.05; anaerobic training: F(2,53) = 1.127, p>0.05; multimodal training: F(2,40) = 0.243, p>0.05; psychomotor training not applicable). For healthy older adults specifically, there were no significant predictors of effect size (S6 Table). However when only older adults with cognitive impairments were considered session duration and frequency appeared significant predictors (S6 Table). Post-hoc tests revealed that programs with shorter session duration and higher frequency significantly predicted higher effect sizes (Table 5).

**Table 5:** Moderator analyses and mean cognitive effect sizes in the mixed-effects model.

		Total	Healthy	Cognitive impairments	Moderator?		
					Total group	Healthy only (post-hoc)	Cognitive impairments only (post-hoc)
Group					No†	No†	No†
	Healthy	d = 0.26** [0.15, 0.37]	-	-			
	Cognitive impairments	d = 0.22* [0.05, 0.39]	-	-			
Cognitive domain					No†	No†	No†
	Global cognition	d = 0.31** [0.14, 0.49]	d = 0.17 [-0.13, 0.46]	d = 0.37** [0.15, 0.60]			
	Executive function	d = 0.25** [0.14, 0.36]	d = 0.27** [0.15, 0.40]	d = 0.16 [-0.11, 0.43]			
	Memory	d = 0.19** [0.05, 0,.32]	d = 0.24** [0.06, 0.41]	d = 0.09 [-0.11, 0.29]			
Exercise type					No†	No†	No†
	Aerobic	d = 0.20** [0.05, 0.35]	d = 0.22* [0.03, 0.41]	d = 0.22 [-0.20, 0.64]			
	Anaerobic	d = 0.26** [0.09, 0.42]	d = 0.27** [0.09, 0.46]	d = 0.22 [-0.20, 0.64]			
	Multicomponent	d = 0.32** [0.12, 0.53]	d = 0.30 [-0.01, 0.60]	d = 0.36* [0.04, 0.68]			
	Psychomotor	d = 0.28 [-0.20, 0,76]	d = 0.29 [-0.21, 0.78]	n/a			
Program duration (weeks)					No†	No†	No†
	Short (4–12)	d = 0.33** [0.18, 0.48]	d = 0.36** [0.17, 0.54]	d = 0.29 [-0.01, 0.58]			
	Moderate (13–24)	d = 0.21** [0.07, 0.35]	d = 0.14 [-0.04, 0.32]	d = 0.34 [0.05, 0.53]			
	Long (≥24)	d = 0.17* [0.01, 0.34]	d = 0.29* [0.06, 0.52]	d = 0.10 [-0.16, 0.36]			
Session duration (minutes)							Yes: F(2,51) = 5.756, p≤0.01**
	Short (≤30)	d = 0.33** [0.14, 0.53]	d = 0.22 [-0.03, 0.47]	d = 0.43 [0.24, 0.62]			β = 0.38, 95%CI [0.15, 0.60]**
	Moderate (31–45)	d = 0.14 [-0.11, 0.38]	d = 0.11 [-0.16, 0.38]	d = 0.28 [-0.26, 0.82]			β = 0.23, 95%CI [-0.33, 0.78]
	Long (≥45)	d = 0.24** [0.12, 0.36]	d = 0.31** [0.16, 0.45]	d = 0.05 [-0.07, 0.17]			Reference
Frequency (#week)					No†	No†	Yes: F(2,51) = 3.589, p≤0.05*
	1/week	d = 0.32 [-0.03, 0.68]	d = 0.23 [-0.17, 0.63]	n/a			n/a
	2/week	d = 0.18* [0.01, 0.36]	d = 0.34* [0.07, 0.62]	d = 0.05 [-0.15, 0.26]			Reference
	3/week	d = 0.25** [0.13, 0.37]	d = 0.23** [0.09, 0.37]	d = 0.35* [0.04, 0.66]			β = 0.22, 95%CI [-0.16, 0.61]
	≥4/week	d = 0.51** [0.24, 0.78]	d = 0.41 [-0.19, 1.00]	d = 0.50** [0.24, 0.76]			β = 0.42, 95%CI [0.06, 0.78]*
Total exercise duration (minutes)		β = 0.00 [-0.00, 0.00]	β = 0.00 [-0.00, 0.00]	β = -0.00 [-0.00, 0.00]	No†	No†	No†
Intensity							
Aerobic exercise	Low intensity 30–50^b^	d = 0.52* [0.09, 0.95]	d = 0.65^c^ [-0.32, 1.62]	d = 0.42 [-0.10, 0.93]	No†	No†	No†
	Moderate intensity 51–80^b^	d = 0.20** [0.06, 0.34]	d = 0.25** [0.07, 0.43]	d = 0.06 [-0.12, 0.24]			
	High intensity 81–100^b^	d = -0.30 [-0.92, 0.32]	d = -0.30 [-0.94, 0.34]	-			
Anaerobic exercise	Low intensity Tertile 1 (i≤1260^a^)	d = 0.13* [0.02, 0.24]	d = 0.19 [-0.01, 0.40]	d = 0.14 [-0.03, 0.31]	No†	No†	No†
	Moderate intensity Tertile 2 (i = 1261–1800^a^)	d = 0.33* [0.03, 0.63]	d = 0.33 [-0.03, 0.69]	-			
	High intensity Tertile 3 (i≥1800^a^)	d = 0.23** [0.07, 0.40]	d = 0.24* [0.03, 0.44]	-			
Multicomponent exercise	Low intensity Tertile 1 (i≤63^a^)	d = 0.47 [-0.32, 1.26]	d = 0.03 [-1.77, 1.84]	d = 1.02 [-0.35, 2.39]^c^	No†	No†	No†
	Moderate intensity Tertile 2 (i = 64–610^a^)	d = 0.22 [-0.30, 0.74]	d = 0.24 [-1.57, 2.04]	d = 0.21 [-0.47, 0.89]			
	High intensity Tertile 3 (i≥611^a^)	d = 0.47 [-0.17, 1.10]	d = 0.49 [-0.80, 1.79]	d = 0.42 [-0.80, 1.64]			
Psychomotor exercise	Low intensity 30–50^b^	d = 0.29 [-0.32, 0.90]	d = 0.29 [-0.32, 0.90]	-	No†	No†	No†
	Moderate intensity 51–80^b^	-	-	-			
	High intensity 81–100^b^	-	-	-			

^a^Arbitrary unit.

^b^%HRR/HRmax/VO2max.

^c^N = 1.

*significant from 0 at p≤0.05;

**significant from 0 at p≤0.01.

^†^p-value >0.05, see text and S6 Table for details.

## Discussion

### Summary of results

We examined the dose-response relationship between a broad sampling of exercises and cognitive function in older adults with and without cognitive impairments. In healthy older adults, there was a small positive effect of exercise on executive function and memory, but not global cognition. In older adults with cognitive impairments, exercise had a moderate positive effect on global cognition, but not executive function or memory. For healthy older adults, there were no significant dose-predictors of cognitive effect sizes. For older adults with cognitive impairments only, programs with shorter sessions and higher frequencies predicted higher cognitive effect sizes.

### Relationship between exercise dose and cognition in healthy older adults

Although exercise carried some beneficial effects for executive function (d = 0.25) and memory (d = 0.24) in healthy older adults, these effects were small and not dose-dependent. The finding that exercise was positively related to executive function and memory in healthy older adults is in line with previous studies in healthy older populations [87–89]. Beneficial effects of exercise on executive function and memory may be fueled by exercise-induced increases in functional connectivity [90], up-regulation of BDNF [89], neocortical modifications [87], and increases in predominantly left hippocampal volume (see [91] for a review). Because optimal executive function and memory are a prerequisite for performing ADLs, the data supports current recommendations for an active lifestyle throughout old age [36].

We found lower exercise effects on cognition in healthy older adults (d = 0.17–0.27) compared with other reviews [33,92], which could be due to the inclusion of only studies that specified all dose-parameters including intensity. Indeed, Northey et al. [33] showed a lower mean cognitive effect when only studies that specified exercise intensity were included in the analysis (d = 0.10–0.16) vs. when all studies were included (d = 0.09–0.69). It is possible that studies that specify dose-parameters are better controlled, yielding smaller effects [93]. This speculation is supported by the small inverse correlation (r = -0.200) between study quality and effect size in our review.

Contrary to meta-analyses showing beneficial effects of longer program duration and higher exercise intensity on physical fitness-parameters [16–18], program duration and intensity did not predict cognitive effects in our review. The finding that longer program duration was not predictive of more cognitive effects is in line with Northey et al. [33]. Although changes in physical fitness-parameters may predict brain plasticity changes, these may not always translate to cognitive benefits [28]. A threshold at which cognitive changes occur is yet to be defined by future studies and can help determine the optimal exercise dose for cognitive improvements. With regards to program duration, the majority of studies in our review reported interventions ≤6 months, and only 3 interventions lasted >6 (i.e., 12) months. Possibly, 6–12 months of exercise is not enough to elicit detectable cognitive effects, considering that early signs of neurodegeneration may emerge in many healthily aging individuals [94]. A lack of intensity effects may stem from heterogeneity sources between the included studies. Some forms of training might be more efficacious than others for physical and cognitive benefits, even when performed at equivalent intensities. For example, walking may be more efficacious than (stationary) cycling at the same intensity, as walking involves the transport of body mass, increasing muscle energy expenditure [95]. Consequently, variation in types of training within exercise categories may have confounded intensity effects. Furthermore, there were differences in intensity measures between studies. For example, aerobic intensity could either be reported in %HRmax, %HRR or %VO2max and we were unable to differentiate between them when calculating the dose-coefficients. This may have inflated the variance in intensity-coefficients. Heterogeneity sources may also have confounded relationships between total dose and the other dose-parameters with cognition. Differences in inclusion criteria yielded variations in the baseline levels of physical activity (PA) of the participants: in four studies [49,51,52,82] only sedentary participants were included, whereas the other studies did not focus on sedentary adults (only). A generally low level of PA has previously been linked to suboptimal cognitive function [96,97]. Participants with lower levels of physical activity may show greater responsiveness to exercise. As PA baseline differences were unaccounted for in the current analyses, they may have confounded a potential dose-response relationship. In addition, differences in cognitive measures may greatly influence the magnitude of the effect. The relationship between exercise dose-parameters and cognitive effects may strongly rely upon the cognitive task difficulty [29,98]. In the current review, we grouped the cognitive tests in three domains: global cognition, executive function, and memory. This may have inflated the variance in effect sizes, potentially diminishing a dose-response relationship between exercise and cognition. To conclude, it is conceivable that the many sources of heterogeneity in the current sample prevented the discovery of an exercise dose-effect on cognition in healthy older adults.

### Dose-response association between exercise and cognition in older populations with cognitive impairments

In older adults with cognitive impairments, exercise had a significant but small positive effect on global cognition (d = 0.37). The effect of exercise on global cognition appeared to stem predominantly from multicomponent training programs. There are a few reasons why multicomponent exercise may be more beneficial than single-modality training. Aerobic and resistance training each may be associated with favorable changes in neurobiological mechanisms (e.g., BDNF, IGF-1, VEGF, homocysteine) [3,5,11,99]. Such changes likely complement each other when aerobic and resistance exercises are performed simultaneously. Also, adding resistance exercise to aerobic training may enhance the neuromotor training stimulus, thereby enhancing cognitive benefits. There is some evidence that adding a balance component to aerobic and strength exercise could result in greater EF and visuospatial ability improvements [100].

Shorter session duration and higher frequency predicted greater cognitive effects. Short sessions may induce less fatigue, which can positively impact the ability and motivation to exercise. High session frequency may decrease overall sedentary time and stabilize levels of exercise-induced neurobiological factors, thereby improving neurocognitive health. However, the relationships between exercise-induced neurobiological mechanisms and dose-parameters are yet to be elucidated in future studies. Last, low session duration and high frequency were mainly evident in shorter programs. This attests for a significant confounding role of life-events during longer exercise programs for patients with cognitive impairments. Additionally, dementia decline may become more pronounced in longer programs. Although a short-term exercise program with low session duration and high frequency may convince patients of the beneficial effects of exercise, it is unlikely that initial effects persist after an intervention. Structural embedding of exercise may be necessary to maintain cognitive function in patients with cognitive impairments. The absence of a relationship between total exercise duration and cognition likely results from a counterbalancing effect between short session duration and higher frequency.

In older adults with cognitive impairments especially, there is a paucity of data concerning exercise dose-parameters and effects on cognitive function. In the current review, only one study compared different exercise doses (lower (40%HRR) vs. higher (60%HRR) intensity cycling) on cognitive function [83]. A lack of intensity effects in the present review could be explained by additional sources of heterogeneity discussed for healthy older adults. First, there were fewer studies in older adults with cognitive impairments. Second, although there was less variation in the cognitive tests used, cognitive test performance variation is generally larger in this population. Thus, the cognitive measures remain a source of large heterogeneity. Third, activities pursued by the control group may affect effect size. Control group activities may be beneficial to cognition for participants who are at high risk of cognitive decline [101]. Future research should investigate whether physical activity is preferable to other activities in improving cognition in older patients with cognitive impairments.

### Limitations

The statistical power of the current analyses is limited by the high level of heterogeneity between studies, dependencies between studies that reported on the same samples, multiple testing and the relative low number of studies in each model. Current results should be carefully interpreted in light of potential type I error inflation, especially when taking into account the additional possible effect due to multiple comparisons. It must be noted that, while significant (d = 0.25–0.37, p<0.05), the small exercise effects on cognition may be of limited clinical relevance. We are uncertain how much change on our composite cognitive domains reflects a clinically relevant change, as we lack observational data linking cognitive changes to health outcomes in our review. Another important limitation is the unclear weighting with which each exercise parameter contributes to the exercise effects and a possible dose-response relationship. We weighted the parameters program duration, session duration, frequency and intensity equally in the determination of exercise dose. However, evidence for such an assumption is lacking because there are no studies that examined the unique contribution of each exercise parameter in isolation. We also assumed that if dose-parameters were not specified in a paper, that the specific range of dose-parameters was not recorded during a trial. It is possible that we have wrongfully excluded some studies because of this assumption. In addition, we cannot confirm the linear or inverted U-shape of the dose-response relationships between exercise and cognition. Furthermore, in the current review, we only included studies where the exact range of dose-parameters was specified. Consequently, we excluded several studies where dose-parameters gradually increased. However, it must be noted that the American College of Sports Medicine [36] specifically encourages a gradual increase of exercise dose-parameters for vulnerable older individuals. Last, the cognitively impaired older groups consisted of older adults with dementia (n = 5 studies), (probable) MCI (n = 6; n = 1 specified as amnestic MCI), VCI (n = 1) or ‘persons with cognitive impairments’ (n = 1). S7 Table shows the verification methods for cognitive status. From a clinical perspective, these syndrome groups are different and we acknowledge the heterogeneity that results from categorizing these subjects as ‘older adults with cognitive impairments’. With respect to MCI, only one study classified subjects with specifically amnestic MCI. There may be differences in brain structure and cognitive function between patients with amnestic vs. non-amnestic MCI [102]. Consequently, exercise may have differential effects on cognition in patients with amnestic vs. non-amnestic MCI, but we were unable to account for such differentiation. In addition, from a clinical perspective, it is valuable to know whether the dose-response relationship between exercise and cognition is different for different syndromes and grades of cognitive pathology. Unfortunately, there are currently not enough studies to provide such information.

### Recommendations for future research

The shortage of studies (six for healthy older adults, one with older adults with cognitive impairments) that compare effects of different exercise doses illustrates the need for within-study variations in dose-parameters, e.g., comparing different exercise doses directly among randomized subjects or conditions allows for a better fit between exercise and its functional benefits for every participant. In addition, the main sources of heterogeneity in the included studies are the types of exercise, research methods, target populations, and cognitive tests. To improve comparison between studies, future studies could reduce such sources of heterogeneity by collecting and reporting as many program-related characteristics as possible, such as a detailed description of the protocol and measures of adherence and compliance.

## Conclusion

The current review cannot confirm nor can reject previously established guidelines on the optimal exercise dose and intensity for healthy older adults. Adhering to these guidelines, older adults should perform a combination of aerobic and anaerobic exercises, of moderate intensity, for at least three times per week, on as many days of the week as feasible [36]. For older adults with cognitive impairments, programs with shorter session duration and higher frequency may generate the best cognitive results. For lasting effects it is recommended to structurally embed exercise in daily life. Health professionals are advised to tailor exercise prescriptions to each individual, as to maximize conformity to exercise programs and ensure long-lasting benefits.

## Supporting information

S1 Checklist PRISMA checklist. (PDF)

S1 Fig Funnel plot. (PDF)

S1 Table Nonspecific search terms. (PDF)

S2 Table Outcome values for studies with healthy old adults. (PDF)

S3 Table Outcome variables for studies with older adults with cognitive impairments. (PDF)

S4 Table Weighted^a^ correlations between dose-parameters and sociodemographic factors for healthy older adults. (PDF)

S5 Table Weighted^a^ correlations between dose-parameters and sociodemographic factors for older adults with cognitive impairments. (PDF)

S6 Table Omnibus tests for the moderator analyses for healthy older adults and older adults with cognitive impairments. (PDF)

S7 Table Verification of cognitive status and years of education. (PDF)
