# Supplementary figures and images for: Dose-response relationship between exercise and cognitive function in older adults with and without cognitive impairment: A systematic review and meta-analysis
Source: PLoS One. 2019 Jan 10;14(1):e0210036. doi: 10.1371/journal.pone.0210036 (PMC6328108; doi:10.1371/journal.pone.0210036)

**S1 Fig. Funnel plot.**

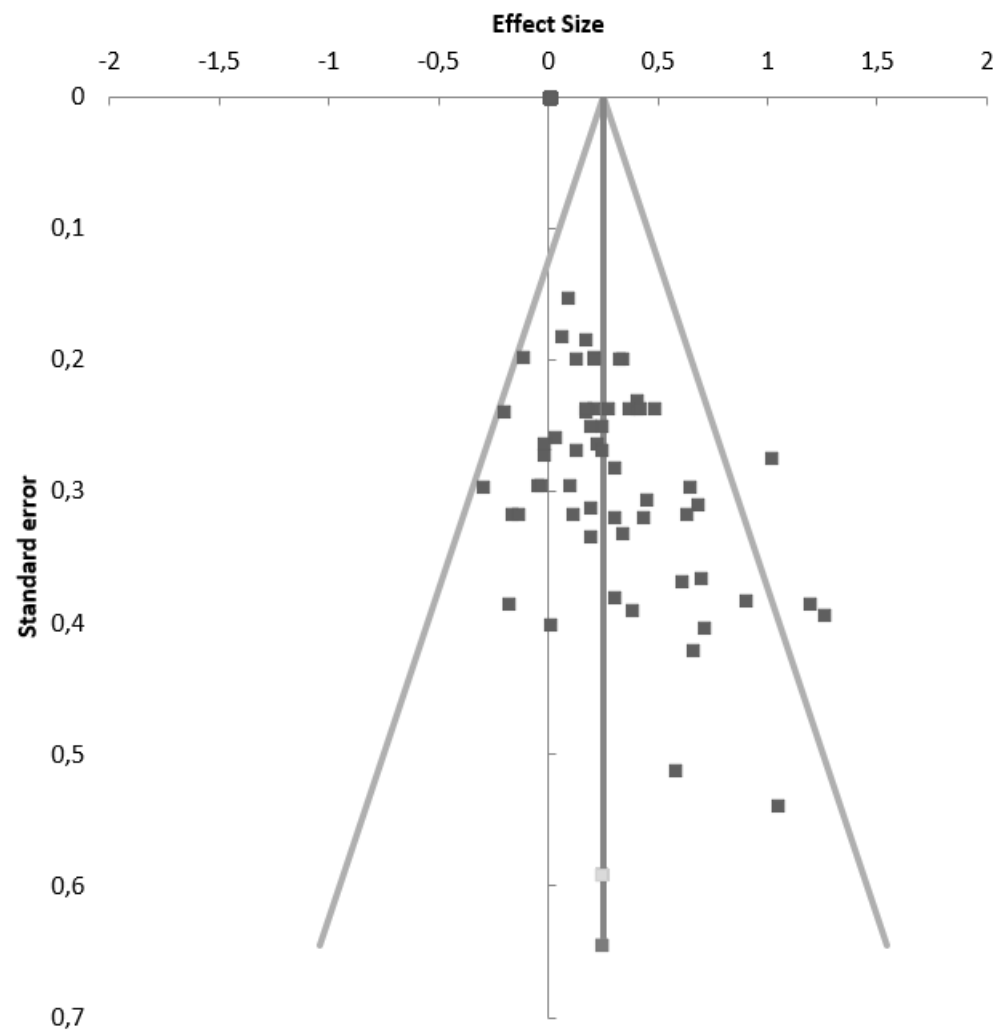

Supplement: S1 Fig — (PDF) [file pone.0210036.s002.pdf]
