# Supplementary material for: Dose-response relationship between exercise and cognitive function in older adults with and without cognitive impairment: A systematic review and meta-analysis
Source: PLoS One. 2019 Jan 10;14(1):e0210036. doi: 10.1371/journal.pone.0210036 (PMC6328108; doi:10.1371/journal.pone.0210036)
Supplement: S1 Table — (PDF) [file pone.0210036.s003.pdf]

S1 Table. Nonspecific search terms.

	Variable	Terms
**Inclusion**	Activity	(exercis* OR physical activit*)
	Training type	(aerobic OR endurance OR anaerobic OR resistance OR strength OR power OR physical OR balance OR psychomotor) AND training
	Cognitive outcome	(cognit* OR cognitive function* OR cognitive performance OR memory OR memory performance OR executive function OR executive process* OR STROOP OR attention OR neurocognit*)
	Study design	(randomised controlled trial OR rct OR controlled)
**Exclusion**	Children or adolescents	(child* OR children OR youth)
	Patient population other than MCI, VCI, dementia	(Parkinson OR MS OR multiple sclerosis OR stroke OR fibromyalgia OR huntington OR cancer OR copd OR asthma OR infarct* OR myocardial infarct* OR schizophrenia OR chronic fatigue syndrome OR diabetes OR retardation OR knee OR knee osteoarthritis OR back pain OR heart failure OR obese)
	Protocol papers	(study protocol OR protocol OR rationale OR design)
	Virtual designs	(virtual)
