# Supplementary material for: Dose-response relationship between exercise and cognitive function in older adults with and without cognitive impairment: A systematic review and meta-analysis
Source: PLoS One. 2019 Jan 10;14(1):e0210036. doi: 10.1371/journal.pone.0210036 (PMC6328108; doi:10.1371/journal.pone.0210036)
Supplement: S2 Table — (PDF) [file pone.0210036.s004.pdf]

S2 Table. Outcome values for studies with healthy old adults.

Author, year	Intervention type (ar/an/mc/pm) [a]	Total exercise duration (min)	Outcome variables	Effect domain[b]	Effect size	PEDro score
Albinet et al., 2010	ar	2160	Wisconsin Card Sorting Task	EF	0,65	4
Albinet et al., 2016	ar	2520	STROOP task; Random Number Generation task; Hayling task; Spatial Running Span task; Verbal Running Span task; 2-back task; Dimension-switching task; Plus-Minus task; Digit-Letter Task	EF	0,19	5
Ansai & Rebelatto, 2015	mc	2880	Montreal Cognitive Assessment	GC	0,09	7
			Clock Drawing Test; Verbal Fluency	EF	-0,31	
	an	2880	Montreal Cognitive Assessment	GC	-0,04	
			Clock Drawing Test; Verbal Fluency	EF	-0,03	
Best et al., 2015	an	3120	Latent memory score	Mem	-0,12	7
			Latent executive function score	EF	0,20	
	an	6240	Latent memory score	Mem	0,32	
			Latent executive function score	EF	0,20	
Coetsee & Terblanche, 2017	ar	1440	STROOP basic, incongruent and interference	EF	-0,31	5
	ar	2256	STROOP basic, incongruent and interference	EF	-0,19	
	an	1440	STROOP basic, incongruent and interference	EF	0,19	
Dao et al., 2013	an	3120	STROOP interference	EF	0,40	3
	an	6240	STROOP interference	EF	0,40	
Fabre et al., 2002	ar	960	Wechsler Memory Scale: total score; Paired associate learning; Logic Memory Immediate Recall; Orientation; General Information; Mental Control; Visual Reproductions	Mem	0,58	5
			Digit Span Forward	EF	1,05	
Ferreira et al., 2015	ar	3960	Wechsler Memory Scale, Logic Memory 1 + 2	Mem	0,45	5
			WAIS, vocabulary + information + symbol search; Digit Span Forward + Backward; Corsi Block Test Forward + Backward; Wisconsin Card Sorting Task, number of categories + perseverative errors	EF	0,68	
Iuliano et al., 2015	an	1080	Attentive Matrices Test; Raven's Progressive Matrices Tests; STROOP; Trail Making Test A + B; Drawing Copy Test, copy with elements	EF	0,30	4

	ar	1080	Attentive Matrices Test; Raven's Progressive Matrices Tests; STROOP; Trail Making Test A + B; Drawing Copy Test, copy with elements	EF	0,43	
	pm	1440	Attentive Matrices Test; Raven's Progressive Matrices Tests; STROOP; Trail Making Test A + B; Drawing Copy Test, copy with elements	EF	0,11	
Jonasson et al., 2017	ar	3240	Word recognition; Free recall, Paired associates	Mem	0,42	5
			D-KEFS Trail Making Test 2+3; Digit Symbol task; Letter comparison; Letter memory; N-back; Keep track; Automated operation span; Flanker task; Backward Digit Span	EF	0,29	
Kimura et al., 2010	an	2160	Task-switching Test	EF	0,16	4
Liu-Ambrose et al., 2010	an	3120	STROOP; Trail Making Test A + B; Digit Span	EF	0,21	7
	an	6240	STROOP; Trail Making Test A + B; Digit Span	EF	0,13	
Liu-Ambrose et al., 2012	an	3120	Flanker task	EF	0,34	6
	an	6240	Flanker task	EF	1,18	
Maass et al., 2015	ar	1440	Verbal Learning and Memory Test early recall, late recall, recognition; Complex figure test early recall; late recall; recognition	Mem	-0,14	5
Madden et al., 1989	ar	2880	Word comparison reaction time task	Mem	-0,03	5
			Letter search reaction time task	EF	0,30	
Muscari et al., 2010	ar	9360	Mini Mental State Examination	GC	0,06	7
Nouchi et al., 2013	mc	360	Verbal fluency task, letterfluency + category fluency; Adapted STROOP task + reverse; Digit Span Forward + Backward; Digit cancellation task; Digit symbol coding; Symbol search	EF	0,25	8
			Logical memory; First and second names	Mem	0,19	
Ruscheweyh et al., 2011	ar	4800	Auditory Verbal Learning Test recall	Mem	0,19	5
	pm	4800	Auditory Verbal Learning Test recall	Mem	0,63	
Shatil et al., 2013	mc	2160	CogniFIT	GC	0,03	4
Tsai et al., 2015	an	9360	Oddball task	EF	0,65	7
Tsai et al., 2017	ar	2880	Mini Mental State Examination	GC	0,37	6
Tsutsumi et al., 1997	an	972	Mental arithmetic task; Computerized mirror drawing task time + distance + errors	EF	0,38	5
	an	1692	Mental arithmetic task; Computerized mirror drawing task time + distance + errors	EF	0,30	

Vedovelli et al., 2017	mc	2160	Digit Span Forward + Backward; Logical Memory 1+2	Mem	1,11	5
			Trail Making Test; STROOP	EF	1,14	

Effect sizes are averages of test-specific effect sizes. [a]ar = aerobic; an = anaerobic; mc = multi-component; pm = psychomotor. [b]GC = global cognition; EF = executive function, Mem = memory.
