# Supplementary material for: Dose-response relationship between exercise and cognitive function in older adults with and without cognitive impairment: A systematic review and meta-analysis
Source: PLoS One. 2019 Jan 10;14(1):e0210036. doi: 10.1371/journal.pone.0210036 (PMC6328108; doi:10.1371/journal.pone.0210036)
Supplement: S3 Table — (PDF) [file pone.0210036.s005.pdf]

S3 Table. Outcome variables for studies with older adults with cognitive impairments.

Author, year	Intervention type (ar/an/mc/pm)[a]	Total exercise duration (min)	Outcome variables	Effect domain[b]	Effect size	PEDro score
Baker et al., 2010	ar	5040	Trail Making Test B; STROOP task; Task Switching; Verbal Fluency; Symbol Digit Modalities	EF	0,71	6
Bossers et al., 2015	mc	1080	Mini Mental State Examination	GC	0,48	6
			Eight-words Test recall + recognition, Digit Span Forward; Visual Memory Span Forward, Rivermead Face + Picture Recognition Test	Mem	0,42	
			Visual Memory Span Backward, Digit Span Backward, STROOP, Verbal Fluency, GIT Picture Completion Test, Trail Making Test A	EF	0,37	
	ar	1080	Mini Mental State Examination	GC	0,21	
			Eight-words Test recall + recognition, Digit Span Forward; Visual Memory Span Forward, Rivermead Face + Picture Recognition Test	Mem	0,28	
			Visual Memory Span Backward, Digit Span Backward, STROOP, Verbal Fluency, GIT Picture Completion Test, Trail Making Test A	EF	0,17	
De Souto Barreto et al., 2017	mc	2880	Mini Mental State Examination	GC	-0,08	7
Ten Brinke et al., 2015	ar	3120	Rey Auditory Verbal Learning Test, total acquisition + recall after interference + loss after interference + long delay free recall + recognition	Mem	-0,18	7
	an	3120	Rey Auditory Verbal Learning Test, total acquisition + recall after interference + loss after interference + long delay free recall + recognition	Mem	0,00	
Davis et al., 2013	ar	2880	STROOP interference	EF	0,21	6
	an	2880	STROOP interference	EF	0,24	
Kemoun et al., 2010	mc	2700	ERFC (Rapid Evaluation of Cognitive Function)	GC	1,25	4
Kwak et al., 2006	an	4550	Mini Mental State Examination	GC	0,89	5
Liu-Ambrose et al., 2016	ar	4320	ADAS-COG	GC	0,16	7
			Trail Making Test; STROOP interference	EF	-0,21	
Nagamatsu et al., 2013	ar	3120	Rey Auditory Verbal Learning Test, total acquisition + recall after interference + loss after interference + long delay free	Mem	-0,02	7

			recall Computerized spatial memory task, one + two + three item reaction time / accuracy + choice reaction time			
	an	3120	Rey Auditory Verbal Learning Test, total acquisition + recall after interference + loss after interference + long delay free recall Computerized spatial memory task, one + two + three item reaction time / accuracy + choice reaction time	Mem	0,13	
Ruiz et al., 2015	mc	1020	Mini Mental State Examination	GC	-0,17	7
Telenius et al., 2015	mc	1320	Mini Mental State Examination	GC	0,09	8
Varela et al., 2011	ar	1080	Mini Mental State Examination	GC	0,69	6
	ar	1080	Mini Mental State Examination	GC	0,61	
Wei & Ji, 2014	mc	3600	Mini Mental State Examination	GC	1,02	5

Effect sizes are averages of test-specific effect sizes. [a]ar = aerobic; an = anaerobic; mc = multi-component; pm = psychomotor. [b]GC = global cognition; EF = executive function, Mem = memory.
