# Supplementary material for: Dose-response relationship between exercise and cognitive function in older adults with and without cognitive impairment: A systematic review and meta-analysis
Source: PLoS One. 2019 Jan 10;14(1):e0210036. doi: 10.1371/journal.pone.0210036 (PMC6328108; doi:10.1371/journal.pone.0210036)
Supplement: S4 Table — (PDF) [file pone.0210036.s006.pdf]

S4 Table. Weighted[a] correlations between dose-parameters and sociodemographic factors for healthy older adults.

	Age	MMSE	Program duration	Session duration	Frequency	Total exercise duration
Age	.	-0.48	-0.05	0.32	0.05	0.05
MMSE	.	.	0.27	0.06	-0.26	0.14
Program duration (weeks)	.	.	.	0.50	-0.69	0.81
Session duration (minutes)	.	.	.	.	-0.54	0.54
Frequency (#/week)	.	.	.	.	.	-0.24
Total exercise duration (minutes)	.	.	.	.	.	.

[a]Correlations weighted for n per study.
