# Supplementary material for: Dose-response relationship between exercise and cognitive function in older adults with and without cognitive impairment: A systematic review and meta-analysis
Source: PLoS One. 2019 Jan 10;14(1):e0210036. doi: 10.1371/journal.pone.0210036 (PMC6328108; doi:10.1371/journal.pone.0210036)
Supplement: S5 Table — (PDF) [file pone.0210036.s007.pdf]

S5 Table. Weighted[a] correlations between dose-parameters and sociodemographic factors for older adults with cognitive impairments.

	Age	MMSE	Program duration	Session duration	Frequency	Total exercise duration
Age	.	-0.92	-0.76	-0.58	0.38	-0.82
MMSE	.	.	0.71	0.69	-0.55	0.74
Program duration (weeks)	.	.	.	0.73	-0.67	0.90
Session duration (minutes)	.	.	.	.	-0.90	0.73
Frequency (#/week)	.	.	.	.	.	-0.48**
Total exercise duration (minutes)	.	.	.	.	.	.

[a]Correlations weighted for n per study.
