# Supplementary material for: Dose-response relationship between exercise and cognitive function in older adults with and without cognitive impairment: A systematic review and meta-analysis
Source: PLoS One. 2019 Jan 10;14(1):e0210036. doi: 10.1371/journal.pone.0210036 (PMC6328108; doi:10.1371/journal.pone.0210036)
Supplement: S6 Table — (PDF) [file pone.0210036.s008.pdf]

S6 Table. Omnibus tests for the moderator analyses for healthy older adults and older adults with cognitive impairments.

	Healthy	Cognitive impairments
Cognitive domain	$F(2,129)=0.313$, $p>0.05$	$F(2,51)=2.050$, $p>0.05$
Exercise type	$F(3,128)=0.081$, $p>0.05$	$F(2,51)=0.432$, $p>0.05$
Program duration (weeks)	$F(2,129)=1.487$, $p>0.05$	$F(2,51)=1.259$, $p>0.05$
Session duration (minutes)	$F(2,129)=0.905$, $p>0.05$	$F(2,51)=5.756$, $p\leq0.01$**
Frequency (#week)	$F(3,128)=0.283$, $p>0.05$	$F(2,51)=3.589$, $p\leq0.05$*
Total exercise duration (minutes)	$F(1,130)=0.365$, $p>0.05$	$F(1,52)=0.000$, $p>0.05$
Intensity[ab]		
Aerobic exercise[b]	$F(2,50)=1.784$, $p>0.05$	$F(1,25)=1.967$, $p>0.05$
Anaerobic exercise[a]	$F(2,35)=0.220$, $p>0.05$	-
Multimodal exercise[a]	$F(2,31)=0.093$, $p>0.05$	$F(2,6)=0.845$, $p>0.05$
Psychomotor exercise[b]	-	-

[a]a.u. = arbitrary unit. [b]%HRR/HRmax/VO2max. *significant from 0 at $p\leq0.05$; **significant from 0 at $p\leq0.01$.
