# Supplementary material for: Dose-response relationship between exercise and cognitive function in older adults with and without cognitive impairment: A systematic review and meta-analysis
Source: PLoS One. 2019 Jan 10;14(1):e0210036. doi: 10.1371/journal.pone.0210036 (PMC6328108; doi:10.1371/journal.pone.0210036)
Supplement: S7 Table — (PDF) [file pone.0210036.s009.pdf]

S7 Table. Verification of cognitive status and years of education.

**Author, year**	**Population**	**Verification of cognitive status or syndrome**	**Years of education (mean±SD) intervention / control**
Albinet et al., 2010	Healthy	Inclusion criterium: MMSE[a]>26	11.3±4.6 / 12.8±3.3
Albinet et al., 2016	Healthy	Inclusion criterium: MMSE[a]>26	11.9±3.9 / 11.6±2.1
Ansai & Rebelatto, 2015	Healthy	Exclusion criterium: MMSE[a] below cut-off designated by educational level – 1 SD	Other
Best et al., 2015	Healthy	Not specified	Other
Coetsee & Terblanche, 2017	Healthy	Exclusion criterium: MoCA[b]<26	Not specified
Dao et al., 2013	Healthy	Inclusion criterium: MMSE[a]≥24	Not specified
Fabre et al., 2002	Healthy	Not specified	11.2±1.3 / 12.1±1.4
Ferreira et al., 2015	Healthy	Inclusion criterium: no mild cognitive impairment or diagnosis of dementia	12.9±2.7 / 12.9±2.5
Iuliano et al., 2015	Healthy	Inclusion criterium: absence of dementia	12.4±3.45 / 12.3±4.30<br>11.1±4.18 / 12.3±4.30<br>11.3±3.88 / 12.3±4.30
Jonasson et al., 2017	Healthy	Exclusion criterium: MoCA[b]<27	Not specified
Kimura et al., 2010	Healthy	Exclusion criterium: diagnosis of dementia by a medical doctor	Not specified
Liu-Ambrose et al., 2010	Healthy	Inclusion criterium: MMSE[a]≥24	Other
Liu-Ambrose et al., 2012	Healthy	Inclusion criterium: MMSE[a]≥24	Other
Maass et al., 2015	Healthy	Exclusion criterium: MMSE[a]≤27	Not specified

Madden et al., 1989	Healthy	Unspecified	15.1±2.06 / 14.9±2.97<br>15.7±2.54 / 14.9±2.97
Muscari et al., 2010	Healthy	Exclusion criterium: MMSE[a]<24	Other
Nouchi et al., 2013	Healthy	Unspecified	13.4±1.85 / 13.2±1.96
Ruscheweyh et al., 2011	Healthy	Exclusion criterium: presence of dementia defined as MMSE[a]<26	10.5±2.8 / 11.0±4.1<br>11.0±2.6 / 11.0±4.1
Shatil et al., 2013	Healthy	Exclusion criterium: MMSE[a]≤23	Other
Tsai et al., 2015	Healthy	Exclusion criterium: MMSE[a]≤26	6.6±2.50 / 6.8±2.71
Tsai et al., 2017	Healthy	Exclusion criterium: MMSE[a]≤26	12.6±2.97 / 10.6±3.20
Tsutsumi et al., 1997	Healthy	Unspecified	Not specified
Vedovelli et al., 2017	Healthy	Exclusion criterium: presence of neurodegenerative diseases; cutoff values for the Brazilian MMSE[a]<18 for middle educational level and <26 for high educational level	7.4±3.39 / 10.1±3.82
Baker et al., 2010	Amnestic MCI	Inclusion criterium: amnestic MCI diagnosis (single or multiple domain) using Petersen criteria	Not specified
Bossers et al., 2015	Dementia	Inclusion criterium: diagnosis of dementia reported in the patient's medical file or a medical specialist	Other
Ten Brinke et al., 2015	Probable MCI	Inclusion criteria: MMSE[a]≥24; MoCA[b]<26; answered "yes" to the question "Do you have any difficulty with your memory?"	Other
Davis et al., 2013	Probable MCI	Inclusion criteria: MoCA[b]<26; presence of subjective memory complaints	Not specified
De Souto Barreto et al., 2017	Dementia	Inclusion criteria: diagnosis of Alzheimer's disease or vascular or mixed dementia according to the Diagnostic and Statistical Manual of Mental Disorders, Fourth Edition (DSM-IV); MMSE[a]≤20	Other
Kemoun et al., 2010	Dementia	Inclusion criteria: diagnosis of Alzheimer-type dementia by a neurologist according to the Diagnostic and Statistical Manual of Mental Disorders, Fourth Edition (DSM IV) criteria; MMSE[a]<23	Not specified

Kwak et al., 2006	Dementia	Inclusion criterium: MMSE[a] 10-26	Not specified
Liu-Ambrose et al., 2016	VCI	Inclusion criterium: clinical diagnosis of VCI based on the presence of small vessel ischemic disease and cognitive syndrome	Other
Nagamatsu et al., 2013	Probable MCI	Inclusion criteria: MMSE[a]≥24; MoCA[b]<26; answered "yes" to the question "Do you have any difficulty with your memory?"	Other
Ruiz et al., 2015	Cognitive impairments (unspecified)	Only 'severe dementia' is an exclusion criteria. However baseline MMSE[a] is 16.4(control)/18.5(intervention), which is indicative of the presence of cognitive impairments	Not specified
Telenius et al., 2015	Dementia	Inclusion criteria: having dementia of mild or moderate degree as measured by the Clinical Dementia Rating scale (CDR 1 or 2)	Not specified
Varela et al., 2011	MCI	Inclusion criterium: confirmed clinical diagnosis of mild cognitive impairment according to the consensus of the Spanish Society of Geriatrics and Gerontology	Not specified
Wei & Ji, 2014	MCI	Inclusion criterium: diagnosis of MCI according to the Diagnostic and Statistical Manual of Mental Disorders, Fourth Edition (DSM IV) criteria; MMSE[a]≤26; Global Deterioration Scale (GDS) score 2-3	Other

[a]Mini-Mental State Examination. [b]Montreal Cognitive Assessment.
